# Functional network alterations differently associated with suicidal ideas and acts in depressed patients: an indirect support to the transition model

**DOI:** 10.1038/s41398-021-01232-x

**Published:** 2021-02-04

**Authors:** Gerd Wagner, Meng Li, Matthew D. Sacchet, Stéphane Richard-Devantoy, Gustavo Turecki, Karl-Jürgen Bär, Ian H. Gotlib, Martin Walter, Fabrice Jollant

**Affiliations:** 1 Department of Psychiatry and Psychotherapy, Jena University Hospital, Philosophenweg 3, 07743 Jena, Germany; 2 Center for Depression, Anxiety, and Stress Research, McLean Hospital, Harvard Medical School, Belmont, MA USA; 3 McGill group for Suicide Studies, McGill University & Douglas Mental Health University Institute, Montréal, QC Canada; 4 Department of Gerontopsychiatry and Psychosomatics, Jena University Hospital, Jena, Germany; 5 Department of Psychology, Stanford University, Stanford, CA USA; 6 Université de Paris, Faculté de médecine, Paris, France; 7 GHU Paris Psychiatrie et Neurosciences, Hôpital Sainte-Anne, Paris, France; 8 Psychiatry Department, CHU Nîmes, Nîmes, France; 9 Equipe Moods, INSERM, UMR-1178 Paris, France

**Keywords:** Human behaviour, Depression, Neuroscience

## Abstract

The transition from suicidal ideas to a suicide act is an important topic of research for the identification of those patients at risk of acting out. We investigated here whether specific brain activity and connectivity measures at rest may be differently associated with suicidal thoughts and behaviors. A large sample of acutely depressed patients with major depressive disorder was recruited in three different centers (Montreal/Canada, Stanford/USA, and Jena/Germany), covering four different phenotypes: patients with a past history of suicide attempt (*n* = 53), patients with current suicidal ideas but no past history of suicide attempt (*n* = 40), patients without current suicidal ideation nor past suicide attempts (*n* = 42), and healthy comparison subjects (*n* = 107). 3-T resting-state functional magnetic resonance imaging (fMRI) measures of the amplitude of low-frequency fluctuation (ALFF) and degree centrality (DC) were obtained and examined in a whole-brain data-driven analysis. Past suicide attempt was associated with a double cortico-subcortical dissociation in ALFF values. Decreased ALFF and DC values mainly in a frontoparietal network and increased ALFF values in some subcortical regions (hippocampus and thalamus) distinguished suicide attempters from suicide ideators, patient controls, and healthy controls. No clear neural differences were identified in relation to suicidal ideas. Suicide attempters appear to be a distinct subgroup of patients with widespread brain alterations in functional activity and connectivity that could represent factors of vulnerability. Our results also indirectly support at the neurobiological level the relevance of the transition model described at the psychological and clinical levels. The brain bases of suicidal ideas occurrence in depressed individuals needs further investigations.

## Introduction

More than 800,000 individuals die from suicide each year in the world and ten to twenty times more attempt suicide^1^. Unfortunately, prediction and prevention of suicidal behavior (SB) are complicated notably due to a lack of valid diagnostic instruments^2^ and insufficient knowledge regarding its complex pathophysiological mechanisms.

Over the past decade, a growing number of neuroimaging studies have been published investigating structural and functional brain abnormalities associated with SB with the aim to provide more objective measures^3,4^. These studies have yielded mixed results that have been explained by heterogeneities across studies in population characteristics, sample size, clinical conditions, acquisition and analysis methods, or age groups, among others. One significant limitation of this literature is that few studies have directly compared brain functioning and structure related to suicidal ideation (SI) and to suicidal acts (SA)^4^.

Indeed, SI is the first step on the pathway to SA but most individuals with SI do not attempt suicide^5^. In addition, clinical risk factors of SI and SA partly overlap but also present some unique features^6–8^. In other words, it is relevant to consider suicidal ideas and suicidal acts as linked but also different phenotypes. A recent study provides evidence that psychological differences between suicide ideators and attempters in clinical variables are particularly complex and that combinations of several factors contribute to the transition from SI to SA^9^. However, this study was based on self-reports, which may have higher variability, be prone to different kind of bias and might be thus less objective than neuroimaging parameters, such as resting-state functional Magnetic Resonance Imaging (rs-fMRI) connectivity/activity^10^. Thus, an important yet understudied research topic is to improve our understanding of the neural basis of the transition from SI to SA. While it is ethically complicated to measure this transition within the same individual, advancing the neuroscience of SI vs. SA may allow to better differentiate neural states associated with the thought process and those associated with action in the context of SI. This may ultimately help to develop algorithms based on neural signatures to identify subjects who are at the highest risk for a suicidal act when SIs are present.

Several previous studies used resting-state functional connectivity and other rs-fMRI measures to map dysfunctional network architectures in suicide attempters^11,12^, suicide ideators^13,14^, and healthy relatives of suicide victims^12^. These studies suggest that functional connectivity is promising in the identification of brain dysfunctions associated with suicidal behavior.

Among various measures, the amplitude of low-frequency fluctuations (ALFF) is an rs-fMRI derived measure that reflects the magnitude of spontaneous blood-oxygen-level-dependent (BOLD) signal and has been shown to be directly correlated with the rate of regional glucose metabolism^15^ as well as with task-based BOLD responses^16^. ALFF has been used to infer brain activity in various neuropsychiatric and neurological disorders^15,17,18^. Additionally, in contrast to the commonly used seed-based functional connectivity (FC) analyses, ALFF metrics can be statistically mapped at the whole-brain level without a priori selection of a seed region, which may improve the comparability of ALFF results across studies and provide more data-driven results than do traditional functional connectivity approaches. Moreover, ALFF provides a proxy measure of brain activity *within* a specific region, rather than an assessment of *interactions between* brain regions, thereby representing a different index of brain functioning than functional connectivity-based approaches.

To our knowledge, only two previous studies have used ALFF to study resting-state brain activity in individuals with and without a past history of SA while controlling for the main diagnosis, here Major Depressive Disorder (MDD)^11,19^. In suicide attempters compared to depressed controls, these studies showed significantly higher ALFF values in the right superior and left middle temporal, and occipital regions as well as significantly lower ALFF values in the left superior and middle frontal gyri.

Furthermore, two recently published studies investigated ALFF in depressed patients with only SI. Higher ALFF values were reported in the right hippocampus, bilaterally in the thalamus and caudate in depressed patients with SI compared to depressed patients without SI^14^. Decreased ALFF-based brain dynamics was observed in the SI group in the left hippocampus, dorsal anterior cingulate cortex, left orbitofrontal cortex, and inferior temporal gyrus compared with the non-SI group^20^. Previous ALFF and seed-based FC studies, therefore, suggest distinct neural patterns in patients with SI only compared to patients with a history of SA. However, these studies investigated relatively small sample sizes and used uncorrected statistical approaches that may have led to false positives. Moreover, none of these studies directly compared depressed patients with SA and with SI only.

Of note, differences in abnormalities in local brain activity between patients and healthy controls do not provide information regarding whether local abnormalities in spontaneous brain fluctuations are associated with divergent connectivity. Thus, in addition to ALFF, it is interesting to use a well-established FC measure called “degree centrality” (DC), which takes into account the relation of a voxel with the entire network connectivity^21^. Increased DC is thought to represent the increased number and strength of the respective region’s functional connectivity, thus indicating the region’s functional significance in the brain. Studying functional connectivity may shed light on previous findings in SA and SI using different seed regions and suggesting that abnormal FC in specific brain networks may be a basis for vulnerability to suicide^22–27^. Like the ALFF approach, DC does not require a priori seed selection and, to our knowledge, a data-driven whole-brain FC analysis in patients with SA or SI was not published yet.

Therefore, to elucidate intrinsic brain activity and connectivity differently associated with SA and SI, and to circumvent the above-mentioned limitations of prior research, the present study used rs-fMRI datasets from three different centers to constitute a large study sample of 135 acutely depressed individuals with various suicidal phenotypes, as well as 107 matched healthy controls. Both ALFF and DC measures were compared between groups. Furthermore, all recruited patients were depressed at the time of scanning and diagnosed with MDD to limit group heterogeneity with regard to alterations in FC^28^ and to control for potential different contributions of psychiatric disorders to suicide risk.

The main objectives of this study were therefore to determine: (i) whether patients with current SI exhibit abnormal brain activity and connectivity compared to patients without SI and without SA and (ii) whether there is overlapping and/or distinct pattern of brain activity and connectivity between SI and SA. Based on previous studies, we hypothesized abnormal brain activity in a network comprising temporal, lateral frontal, and limbic regions, i.e. hippocampus and caudate, which have been shown to be generally associated with SB.

## Materials and methods

### Participants

Participants were recruited in three institutions in Montreal (Quebec, Canada), Stanford (CA, USA), and Jena (Germany) and four groups of male and female participants aged 18–63 years were established (Table 1): (1) currently depressed patients with a past history of SA (*suicide attempters, N* = *53*), (2) currently depressed patients with current SI but no past history of SA (*suicide ideators, N* = *40*), (3) currently depressed patients without current SI nor past history of SA (*patient controls, N* = *42*), and (4) non-depressed controls with no history of MDD, SI or SA (*healthy controls, N* = *107*).

**Table 1:** Demographic and clinical sample characteristics.

	Healthy controls	Patient controls	Suicide ideators	Suicide attempters	*F*/*χ*^2^	*p*	Post-hoc
	*n* = 107	*n* = 42	*n* = 40	*n* = 53			
Gender, N males (%)	39 (36.4)	19 (45.2)	9 (22.5)	16 (15.0)	5.3	n.s.	
Age, mean (SD)	35.3 (9.9)	37.5 (11.8)	37.0 (12.2)	38.0 (11.1)	0.9	n.s.	
BDI score, mean (SD)	1.9 (2.2)	21.6 (7.4)	33.3 (11.7)	28.0 (12.6)	11.6*	<0.001*	SI > PC, SA; SA > PC
HAM-D-17 score, mean (SD)	n.a.	14.6 (7.1)	22.7 (5.4)	21.1 (8.1)	15.8	<0.001*	SI > PC; SA > PC
Antidepressant medication					3.4	n.s.	
yes (%)	0 (0)	18 (42.9%)	22 (55%)	28 (52.8%)			
no (%)	107 (100)	24 (57.1%)	18 (45%)	25 (47.2%)			
Previous suicide attempts, mean (SD)				1.7 (0.5)			
Suicide method*,							
violent (%)				16 (30.2%)			
non-violent (%)				37 (69.8%)			

*Statistical comparisons were made between patient groups only.

As depicted in Table 1, all patients were depressed at the time of scanning, as determined by the 17-item Hamilton Depression Rating Scale (HAM-D-17) and all presented with a diagnosis of major depressive episode according to the Structured Clinical Interview for DSM-IV Axis I Disorders (SCID-I).

SA was defined according to Silverman et al^29^. as a “self-inflicted, potentially injurious behavior with a nonfatal outcome for which there is evidence (either explicit or implicit) of intent to die.”

SIs were identified with a score on item #3 of the HAM-D scale >0 (i.e. scores 1, 2, or 3; a score of 4 was limited to suicide attempt occurrence) **and** a score on the item #9 of the Beck Depression Inventory (BDI) >0, which addresses suicidal thoughts in the 2 weeks before assessment and having a four-level response set, i.e. (0) “I don’t have any thoughts of killing myself,” (1) “I have thoughts of killing myself, but would not carry them out,” (2)“ I would like to kill myself,” and (3) “I would kill myself if I had the chance.” (see Fig. S1 for the distribution of patients on HAM-D item #3 and on the BDI item #9). These items have been used in prior studies for the screening of suicide risk^30–33^.

Exclusion criteria comprised a lifetime history of schizophrenia or bipolar disorder, a history of alcohol/substance abuse or dependence spanning the previous 6 months, a major general medical condition requiring ongoing pharmacological treatment, a lifetime history of severe head trauma or central nervous system disorder, and contraindication to MRI. All participants except nine were right-handed.

This study was approved by the respective local ethics committees. Informed written consent was obtained from all participants prior to their participation. Participants received a total of 100 Canadian dollars in Montreal, 25 US dollars per hour in Stanford, or 10 euros per hour of participation in Jena.

### Image acquisition

In Montreal and Jena, MRI scans were acquired using a Siemens Magnetom Trio (Tim System 3T) MRI scanner; in Stanford, scans were acquired using a General Electric 3T (Discovery MR750) MRI scanner. Participants at all sites were instructed to keep their eyes closed during the data collection. Details about scanner parameters are provided in the **supplementary material**.

### Image preprocessing

The rs-fMRI data were processed using the DPARSF toolbox (http://www.restfmri.net) implemented in MATLAB R2019a and applying standard preprocessing steps (details are provided in **supplementary material**). We did not conduct global signal regression^34^. To account for site-related variation in rs-fMRI metrics, post-hoc standardization of all computed rs-fMRI metrics was performed using group-level mean regression^35^ and *site* was included as a covariate into the ANCOVA model.

### ALFF calculation

The ALFF images were computed by extracting power spectra via a Fast Fourier Transform and computing the sum of amplitudes in the low-frequency bands (0.01–0.1 Hz). Then, ALFF values were transformed into *Z*-scores and smoothed using a 4-mm Gaussian kernel.

### Degree centrality (DC)

For each participant, Pearson’s correlation coefficients were calculated between a given voxel and all other voxels in the brain, resulting in a whole-brain FC matrix. We restricted our analysis to positive correlations above a threshold of *r* = 0.25 to eliminate such voxels having low correlation due to signal noise^21,36^. That is, for a given voxel, the number of voxels was counted where the correlation between that voxel and another voxel’s BOLD time series exceeded the fixed threshold (i.e., *r* > 0.25). Subsequently, these DC maps were *z*-transformed and smoothed using a 4-mm Gaussian kernel.

### Statistical analysis

We first tested for the main effect of group separately, on ALFF and DC values by means of a one-way ANCOVA in SPM12 (https://www.fil.ion.ucl.ac.uk/spm/software/spm12/) with four levels of the factor *group* (healthy controls, patient controls, suicide ideators, suicide attempters) and with three *covariates* (age, gender, and site). The resulting overall F-test was thresholded at an FWE-corrected voxel-level significance of *p* < 0.05 and at an FDR-corrected cluster-level significance of *p* < 0.05 due to concerns related to the validity of cluster inference using FWE correction^37^. The FWE correction in SPM is based on the Random Field Theory (RFT) assuming a Gaussian distribution^38^.

Between groups, *post hoc* analyses were performed within the ANCOVA design. The significant main effect of the *group* regarding ALFF or DC values, respectively, was used as a mask image to perform planned between group comparisons across identified significant voxels at the FWE corrected level. In order not to excessively increase the risk of false negatives, these contrasts were thresholded at an uncorrected voxel-level significance of *p* < 0.001 with a cluster extent of *k* ≥ 10, corresponding to the “expected voxels per cluster” value generated by SPM12. In the result tables, we additionally reported the FWE- and FDR-corrected peak *p* values for the local maxima. To create boxplots, the averaged voxel values from significant clusters were extracted and further processed using IBM SPSS software V21 (https://www.ibm.com/de-de/products/spss-statistics).

## Results

Sociodemographic and clinical characteristics of the four groups are presented in Table 1. Of note, there were no significant differences regarding current antidepressant medication status between the three patient groups. A significant difference between patients with SI only and patients with SA was found regarding the item #9 (“suicidal ideation”) of the BDI questionnaire, (SI: Mean = 1.3, [SD = 0.51]; SA: Mean = 0.92 [SD = 0.97], *t* = 2.1, *p* = 0.04) suggesting higher levels of SI in patients with SI.

### ALFF

#### Main effect of group

**Fig. 1:**
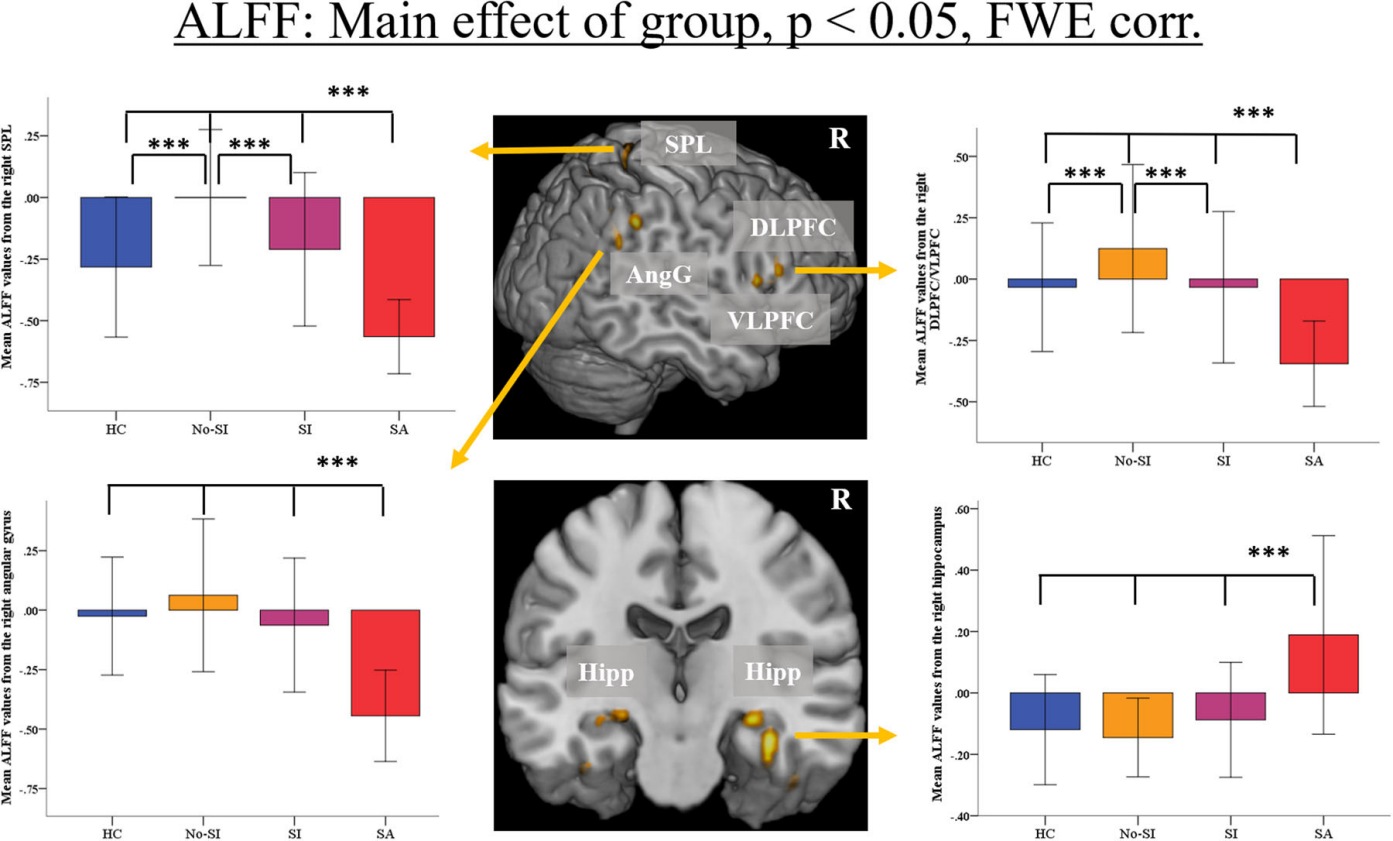
Main effect of group (voxel-level FWE-corrected *p* < 0.05 and cluster-level FDR-corrected *p* < 0.05) on Amplitude of Low-Frequency fluctuations (ALFF). Only selected regions are shown here. The box plots depict mean z-transformed ALFF values as extracted from the significant clusters located in the right angular gyrus (AngG), in the right dorsolateral prefrontal cortex (DLPFC), in the superior parietal cortex (SPL) and the right hippocampus (Hipp). Significant group differences are indicated for the post-hoc comparisons at *p* < 0.001 (***). The error bars represent ± 1 SD. Abbreviations: HC healthy controls, No-SI patients without current suicidal ideations and without past suicide attempt, SI patients patients with current suicidal ideations and without past suicide attempt, SA patients with past suicide attempt, ventrolateral prefrontal cortex (VLPFC).

A significant overall main effect of group (voxel-level FWE-corrected *p* < 0.05, cluster-level FDR-corrected *p* < 0.05) on ALFF values was found in a number of cortical and subcortical regions, including the right dorsolateral (DLPFC) and ventrolateral prefrontal cortex (VLPFC), bilateral parietal (inferior and superior parietal lobules (IPL/SPL), angular gyrus (AngG), precuneus, supramarginal gyrus, post-central gyri), bilateral temporal (fusiform gyrus, superior temporal gyrus), and occipital cortices, bilateral hippocampus and right thalamus (Fig. 1 and Table 2).

**Table 2:** Detailed results of the main effect of group (voxel-level FWE-corrected *p* < 0.05 and cluster-level FDR-corrected *p* < 0.05) on Amplitude of Low-Frequency Fluctuations (ALFF).

Region of activation	Right/Left	Brodmann’s area	Cluster size	MNI coordinates			*F* value
				*x*	*y*	*z*	
Angular gyrus	R	39	74	50	−54	34	24.1
Precuneus	R	7	46	18	−66	44	22.7
Angular gyrus	L	39	40	−46	−62	32	21.8
Inferior parietal cortex	R	40	57	56	−46	40	19.4
Inferior parietal cortex	L	40	31	−56	−42	38	19.3
Hippocampus	R		102	34	−20	−20	19.1
Fusiform gyrus	R	20	14	42	−14	−36	18.6
Cuneus	R	18	20	4	−80	32	18.2
Superior parietal cortex	R	7	21	24	−58	64	17.8
Hippocampus	R		30	28	−20	−12	17.7
Fusiform gyrus	L	20	48	−36	−28	−26	17.6
Postcentral gyrus	R	5	54	18	−44	66	17.2
Inferior parietal cortex	L	40	47	−52	−52	36	16.7
Paracentral lobule	R	4	13	6	−28	66	16.7
Occipital cortex	L	19	16	−40	−74	24	16.4
Superior temporal gyrus	R	22/39	42	58	−30	16	16.2
Inferior frontal gyrus	R	9/45	32	50	24	16	16.0
Supramarginal gyrus	L	39/40	11	−58	−50	24	15.9
Inferior frontal gyrus	R	44	14	54	14	10	15.9
Superior parietal cortex	L	7	22	−26	−64	46	15.6
Cuneus	R	18	33	−2	−86	18	15.6
Fusiform gyrus	L	20	15	−36	−16	−30	15.2
Hippocampus	L		28	−22	−22	−10	15.1
Supramarginal gyrus	R	40	19	60	−30	42	14.8
Superior temporal gyrus	L	22/39	15	−58	−40	20	14.6
Precuneus	L	7	11	−14	−66	58	14.4
Inferior parietal cortex	L	40	49	−54	−32	44	14.4
Occipital cortex	R	19	16	44	−72	12	14.0
Thalamus	R		10	18	−24	20	14.0
Occipital cortex	R	19	14	22	−80	38	13.3

#### Post-hoc analyses

Planned post-hoc analyses showed a pattern of significantly lower ALFF in suicide attempters relative to the three other groups mainly in fronto-parietal regions including the right DLPFC/VLPFC, bilateral AngG and supramarginal gyrus, IPL, SPL, as well as occipital regions (Fig. 1 and Table S1). In addition, suicide attempters showed a pattern of significantly higher ALFF values bilaterally in the hippocampus, in the right thalamus and in the right fusiform gyrus (Fig. 1 and Table S1) compared to suicide ideators, patient controls, and healthy controls.

In addition, patient controls relative to healthy controls exhibited higher ALFF values in right DLPFC, bilateral SPL, right occipital cortex (Fig. 1 and Table S2) and compared to suicide ideators higher values in right SPL, having thus the largest ALFF values in these regions compared to all other groups. There were no further significant differences in the group comparisons.

### DC

#### Main effect of group

**Fig. 2:**
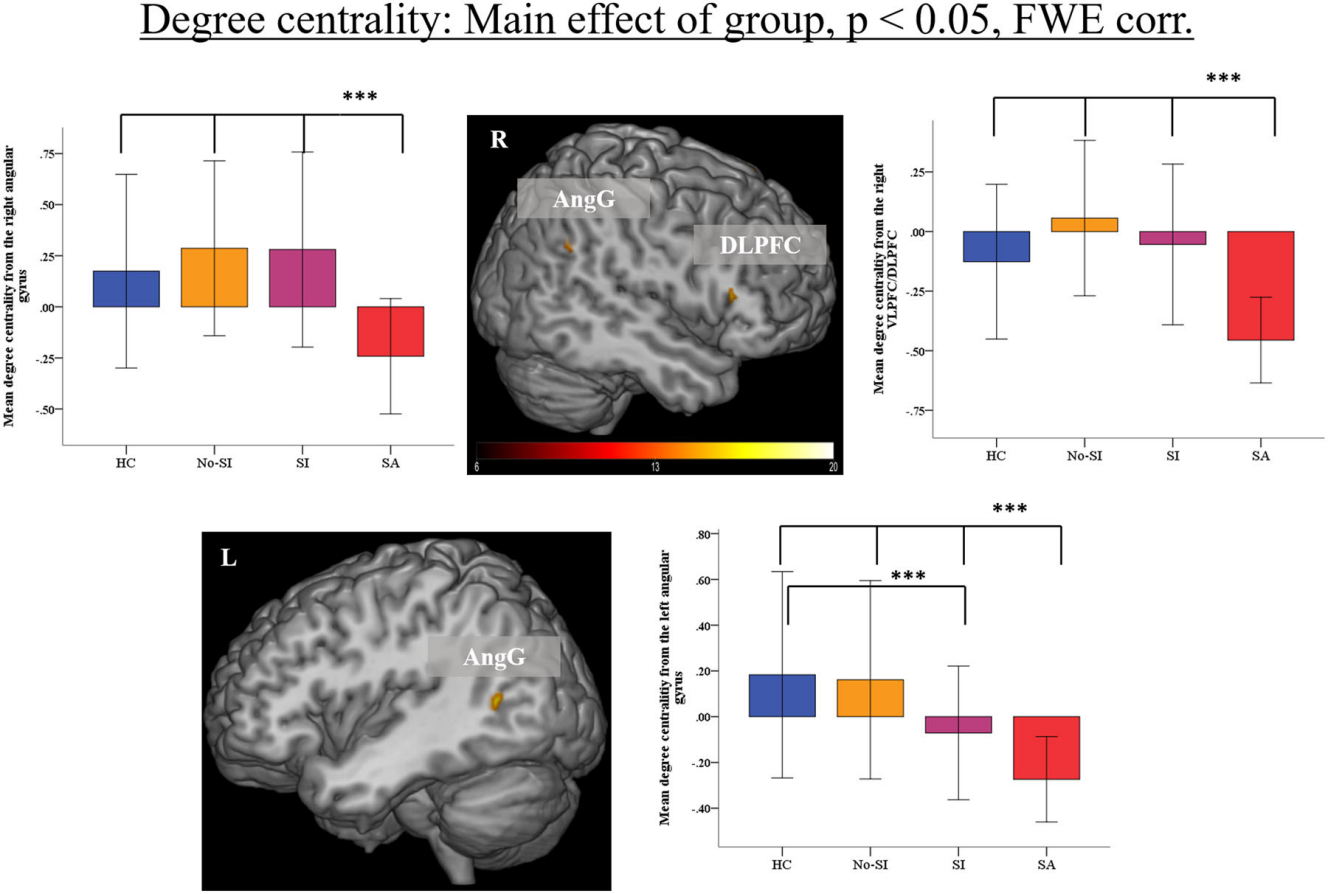
Main effect of group (voxel-level FWE-corrected *p* < 0.05 and cluster-level FDR-corrected *p* < 0.05) on degree centrality (DC). Only selected regions are shown here. Mean z-transformed DC is depicted for the right (AngG, left box plot) and left angular gyrus (AngG, bottom box plot), for the right dorsolateral prefrontal cortex (DLPFC, right box plot). Significant group differences are indicated for the post-hoc comparisons at *p* < 0.001 (***). The error bars represent ± 1 SD. Abbreviations: HC healthy controls, No-SI patients without current suicidal ideations and without past suicide attempt, SI patients patients with current suicidal ideations and without past suicide attempt, SA patients with past suicide attempt.

As depicted in the Fig. 2 and Table 3, a significant overall main effect of group (voxel-level FWE-corrected *p* < 0.05, cluster-level FDR-corrected *p* < 0.05) on DC values was found in similar regions as for ALFF, mainly frontoparietal cortical regions including the right DLPFC/VLPFC and SPL, bilaterally the AngG and occipital cortex.

**Table 3:** Detailed results of the main effect of group (voxel-level FWE-corrected *p* < 0.05 and cluster-level FDR-corrected *p* < 0.05) on Degree Centrality (DC).

Region of activation	Right/Left	Brodmann’s area	Cluster size	MNI coordinates			*F* value
				*x*	*y*	*z*	
Superior parietal cortex	R	7	31	18	−66	42	25.5
Angular gyrus	L	39	36	−42	−60	10	18.8
Occipital cortex	L	18	14	−14	−72	−2	17.2
Angular gyrus	R	39	16	46	−54	36	16.3
Occipital cortex	R	18	23	20	−76	22	15.6
Inferior frontal gyrus	R	9/45	11	48	24	12	15.2
Occipital cortex	L	19	12	−20	−82	24	13.3

#### Post-hoc analyses

Planned post-hoc analyses showed a consistent pattern of significantly lower DC in suicide attempters in comparison to the three other groups in the right DLPFC/VLPFC, bilateral AngG, right SPL, and left occipital cortex (Table S3 and Fig. 2). No voxels with significantly higher DC values were observed in suicide attempters compared to other three groups. In addition, we found that suicide ideators showed reduced DC in the left AngG compared to healthy controls (Table S3 and Fig. 2). Suicide ideators showed also reduced DC compared to patient controls in the same region (MNI coordinates: *x* = −42, *y* = −56, *z* = 6, *t* = 5.1, *p* < 0.001, uncorr., cluster size = 8), but only at an uncorrected voxel-level threshold and with cluster extent of *k* < 10. Patient controls exhibited higher DC values than suicide ideators in the occipital cortex. Furthermore, we found that both, patient controls as well as suicide ideators had significantly higher DC values in the right SPL compared to healthy controls (Table S3).

### Correlations

Using multiple regression analysis in SPM12, no significant correlations were found between significant voxels in the main effect of group contrast on ALFF or DC values and depression severity in patient groups altogether. In addition, we did not find any significant correlation between the number of suicide attempts and altered ALFF or DC values, respectively.

### Site effects

Even if we controlled for potential effects of site on the results by a post-hoc standardization regression^35^ and by using *site* as a covariate in the ANCOVA analyses, we additionally examined potential scanner effects by comparing healthy subjects, which constituted the largest sample (Stanford: *n* = 36; Jena: *n* = 29 and Montreal: *n* = 42), from the three different sites. Using ANCOVA design controlled for age and gender, significant (voxel-level: *p* < 0.05, FWE corrected, cluster-level: *p* < 0.05 FDR-corrected) the main effect of *site* on ALLF values was mainly detected in voxels located in the cerebrospinal fluid, white matter, and dorsal brainstem close to the fourth ventricle (see Fig. S2). Finally, we tested whether the above reported significant results are within this statistical map of site differences and did not find any significant voxels (voxel-level: *p* < 0.05, FWE corrected, cluster-level: *p* < 0.05 FDR-corrected).

## Discussion

In the present study intrinsic whole-brain, resting-state brain activity and connectivity were used to investigate different neural mechanisms associated with the occurrence of SI and SA in depressed patients. To this aim, we compared four groups of individuals with various suicidal phenotypes. By using multicenter fMRI datasets that were relatively large and balanced in terms of sample sizes per group, we thus circumvented the problem of low statistical power that has limited previous studies. Regarding our main objectives, we did not find clear neural differences in relation to SI compared to non-suicidal depressive state. In contrast, our results provide new evidence in support of suicide attempters as a distinct sub-sample of patients with significant neural differences in comparison to patients with SI and to non-suicidal patients.

More specifically, a history of SA was associated with a double cortico-subcortical dissociation in ALFF/DC metrics. Decreased ALFF and DC values mainly in a frontoparietal network and increased ALFF values mainly in subcortical regions (thalamus and hippocampus) distinguished suicide attempters (who were also experiencing current SI) from all other groups, notable patients with current SI who never attempted suicide. These results partly confirm findings from the few previous studies that have used ALFF to map the brains of depressed patients with a past history of SA and reported significantly lower ALFF values in the lateral PFC and occipital cortex in suicide attempters compared to depressed controls^19^. Consistent with DC results from the current study, a recent study reported decreased functional connectivity in depressed patients with SA in contrast to patients with SI and to patient controls without SI and past SA in several frontoparietal regions, including the right DLPFC/VLPFC and the right precuneus^39^.

In our recent rs-fMRI connectomics study, decreased FC in patients with past SA was observed in a set of regions comprising inferior frontal, parahippocampal, as well as somatosensory-motor, superior parietal, and occipital regions compared to healthy controls^12^. Similar results were found in healthy relatives of suicide victims in the same study, suggesting a potential heritability of fronto-parietal BOLD signal abnormalities. Moreover, regions with observed differences in ALFF and DC in suicide attempters overlap with previously identified brain regions from studies that have used structural neuroimaging techniques^40–43^, even if newer studies and meta-analysis could not replicate these findings in general^4,44,45^. Furthermore, previous post-mortem studies in patients with MDD—most of them who died from suicide—have found histopathological abnormalities predominantly in the DLPFC and VLPFC in terms of decreased cell size and density^46–48^.

Regions with abnormal ALFF and DC values identified in our study also overlap with brain deficits observed in task-based functional neuroimaging and associated with specific neurocognitive alterations that could play a significant role in the suicidal transition^49,50^, notably risky decision-making^50,51^, and reduced cognitive control^52^. Altered decision-making processes were associated with abnormal activation in the ventral and dorsal lateral prefrontal cortex in both suicide attempters^51,53,54^ and first-degree biological relatives of suicide completers who themselves never attempted suicide^55,56^. During the classic Go-NoGo task, impaired activation in depressed suicide attempters (although non-specific among depressed patients) was observed in inferior frontal gyrus and parietal cortex^57^. Based on studies in healthy controls, normal cognitive control functions have been consistently associated with activation of a frontoparietal network^58^. Finally, the experience of social rejection, a significant proximal event associated with the risk of suicide, has been associated with a differential activation in the supramarginal gyrus in patients with SA^59^.

In sum, these results suggest that abnormal brain connectivity between frontoparietal regions and subcortical structures may enhance the likelihood that SI is translated into SA. The present study, therefore, expands on prior work by demonstrating that suicide attempters can be differentiated from ideators and non-suicidal patients on deficits in global brain activity and connectivity.

One of the most significant findings regarding abnormal ALFF and DC in our study was found bilaterally in the AngG in patients with SA relative to all other groups. Previous studies in monkeys and humans showed dense anatomical connections between AngG and precuneus/SPL and supramarginal gyrus^60^, as well as with parahippocampal gyrus and hippocampus^61,62^. In all these regions patients with past SA exhibited abnormal brain activity. Interestingly, AngG has also direct and dense connection to the inferior frontal gyrus (BA 44/45)^63^, both regions displaying abnormal ALFF and DC values in patients with SA relative to other groups in our study. Thus, due to this rich anatomical connectivity, it is not surprising that AngG has been shown to be involved in different cognitive/affective/behavioral functions previously associated with a history of SA, including memory retrieval, self-processing, default-mode functions, cognitive control, emotion regulation, and social cognition^3,52,57,62,64^. AngG is therefore considered as a cross-modal hub of several brain networks underlying these various functions. Additional important hubs, whose connectivity appears to be altered in suicide attempters in our study, which may underlie various cognitive alterations, are the DLPFC, superior parietal cortex, and precuneus^65^. Damages to such hubs may impact the efficient information integration between networks more severely than damages to other nodes^66^. In our previous study, we could demonstrate a disorganization in the so-called “rich-club” organization in relation to suicidal behavior, which represents the density of connections between high-degree nodes^67^.

An intriguing finding was that patients with SI showed lower degree connectivity in the left AngG relative to healthy controls and patient controls, indicating a potential continuum between SI and SA. Thus, further studies have to investigate the potential role of this region in the transition from SI to SA, and the possibility that it represents a neural marker of the transition risk.

In addition, we observed ALFF differences in the superior parietal cortex in patients with SI compared to patient controls, but not healthy controls, mainly due to higher ALFF values in patient controls. This is also the case for the inferior frontal gyrus and occipital cortex. These findings are difficult to interpret. However, previous studies that have investigated ALFF in patients with MDD irrespective of SA or SI have reported significant differences between patients and healthy controls in the same regions^68,69^. Greater, as well as lower ALFF values in frontal, striatal, and temporal regions, were found^68^. An explanation may be that alterations are in opposite directions between non-suicidal and suicidal phenotypes, with healthy controls in an intermediate position and the suicidal phenotypes showing an additional continuum of alterations from ideation to acts. This opposite pattern of neuroimaging alterations has previously been observed between patient controls and suicide attempters (with healthy controls in an intermediate position) in a task-based study^70^. Further explanations may be a selection bias in either group. Alternatively, increased activity in patients without SI might indicate a protective factor, which prevent these subjects from experiencing SI while experiencing other depressive symptoms. However, more studies will be necessary to fully clarify the neural basis of SI.

### Limitations

A significant limitation of this study is the reliance on single items from depression rating scales, i.e. HAM-D and BDI to define the SI group, which is heterogeneous and represents a group with relatively less-intense suicidal thinking compared to other studies that have defined this group more rigorously. Future studies should identify an SI group with more severe suicidal plans and intent who have resisted the urge to act, to gain greater insight into the underlying neural mechanisms that prevent or facilitate suicide attempt. Furthermore, differences in fMRI data acquisition hardware and scan parameters between centers may have influenced the reported results. By using the post-hoc standardization approach means of group-level mean regression^35^ and an ANCOVA design with the site as a covariate and conservative statistical threshold, we believe that the influence of site is negligible.

### In conclusion

We showed here that patients with a history of SA (and current SI) are different in terms of at-rest brain functioning than patients with current SI but no past history of SA, with decreased ALFF values and whole-brain connectivity in a network of mainly frontoparietal regions and increased ALFF values in subcortical regions. The risk of SA in patients with SI may therefore result from widespread dysfunctions in brain activity and connectivity including abnormal functioning of specific hubs in the brain, such as of the AngG and others. Our results indirectly confirm at the brain network level the relevance of the transition model described at the psychological and clinical levels. No clear and specific neural differences were identified in relation to SI in the present study, which should prompt further investigations.

## Supplementary information

Supplementary material

Supplementary Figure S1

Supplementary Figure S2

Supplementary Table S1

Supplementary Table S2

Supplementary Table S3
