# Supplementary material for: Functional network alterations differently associated with suicidal ideas and acts in depressed patients: an indirect support to the transition model
Source: Transl Psychiatry. 2021 Feb 4;11:100. doi: 10.1038/s41398-021-01232-x (PMC7862288; doi:10.1038/s41398-021-01232-x)
Supplement: Supplementary file 1 — Supplementary material [file 41398_2021_1232_MOESM1_ESM.docx]

**Functional network alterations differently associated with suicidal ideas and acts in depressed patients: an indirect support to the transition model.**

Gerd Wagner PhD ^1^, Meng Li^1^ PhD, Matthew D. Sacchet MD ^9^, Stéphane Richard-Devantoy MD PhD ^3^, Gustavo Turecki MD PhD ^3^, Karl-Jürgen Bär MD ^2^, Ian H. Gotlib PhD ^8^, Martin Walter MD ^1^, Fabrice Jollant MD PhD ^3,4,5,6,7^.

^1^ Department of Psychiatry and Psychotherapy, Jena University Hospital, Philosophenweg 3, 07743 Jena, Germany;

^2^ Department of Gerontopsychiatry and Psychosomatics, Jena University Hospital, Jena, Germany;

^3^ McGill group for Suicide Studies, McGill University & Douglas Mental Health University Institute, Montréal, Canada;

^4^ Université de Paris, Faculté de médecine, Paris, France ;

^5^ GHU Paris Psychiatrie et Neurosciences, Hôpital Sainte-Anne, Paris, France;

^6^ Psychiatry Department, CHU Nîmes, Nîmes, France;

^7^ Equipe Moods, INSERM UMR-1178, Paris, France

^8^ Department of Psychology, Stanford University, Stanford, CA, USA;

^9^ Center for Depression, Anxiety, and Stress Research, McLean Hospital, Harvard Medical School, Belmont, MA, USA;

**Supplemental information**

**Methods:**

*Image acquisition*

In Montreal and Jena, MRI scans were acquired using a Siemens Magnetom Trio (Tim System 3T) MRI scanner; in Stanford, scans were acquired using a General Electric 3T (Discovery MR750) MRI scanner. Participants at all sites were instructed to keep their eyes closed during the data collection. In Montreal, T2*-weighted images were obtained using a gradient-echo EPI sequence (TR =2090 ms, TE =30 ms, flip angle 90°) with 38 contiguous transverse slices of 3.5 mm thickness and an in-plane resolution of 3.5 × 3.5 mm². A series of 285 whole-brain volume set was acquired in one session. In Jena, T2*-weighted images were obtained using a gradient-echo EPI sequence (TR=2520 ms, TE =30 ms, flip angle 90°) with 45 contiguous transverse slices of 2.5 mm thickness and an in-plane resolution of 2.5 × 2.5 mm² covering the entire brain. A series of 240 whole-brain volume set was acquired in one session. In Stanford T2*-weighted images were obtained using a gradient-echo EPI sequence (TR =2000 ms, TE =30 ms, flip angle 90°) with 42 contiguous transverse slices of 2.9 mm thickness and an in-plane resolution of 2.9 × 2.9 mm² covering the entire brain. A series of 240 whole-brain volume sets was acquired in one session.

High-resolution, whole-brain T1-weighted acquisition was collected using a magnetization prepared rapid gradient echo (MPRAGE) sequence with 1mm³ isotropic voxels in Montreal and Jena, and using whole-brain spoiled gradient echo (spoiled-GE) with an in-plane resolution 0.938 × 0.938 × 1mm³ in Stanford. All scans were inspected for motion artefacts, and a neuroradiologist confirmed the absence of gross pathological findings (except in Montreal).

*Image preprocessing*

The rs-fMRI data were processed using the DPARSF toolbox (http://www.restfmri.net, version 4.5) implemented in MATLAB R2019a. The first ten time-points were discarded to ensure signal stabilization. Slice timing and head motion correction were conducted. For the latter step we used the higher-order regression model, i.e. the Friston 24-parameter model, to regress out head motion effects from the realigned data (6 head motion parameters, 6 head motion parameters one time point before, and the 12 corresponding squared items) [1]. Individual structural T1-weighted images were co-registered to the mean functional image after realignment. The registered structural images were then segmented into gray matter (GM), white matter (WM) and cerebrospinal fluid (CSF). DARTEL was used to compute non-linear transformations of registered T1w images from individual native space to MNI space. The functional images were subsequently normalized to the standard Montreal Neurological Institute template and spatially resampled to a voxel size of 2 × 2 × 2 mm^3^. Linear and quadratic trends were removed, temporal filtering (0.01–0.1 Hz) was subsequently performed on time series except for ALFF metric. Average time courses from the CSF and WM were used as nuisance covariates. We did not conduct global signal regression[2]. To account for site-related variation in rs-fMRI metrics, post-hoc standardization of all computed rs-fMRI metrics was performed using group-level mean regression as described by Yan et al. [3].

*ALFF calculation*

The ALFF images were computed using the DPARSF toolbox by extracting power spectra via a Fast Fourier Transform and computing the sum of amplitudes in the low-frequency bands (0.01–0.1 Hz). Then ALFF was transformed into *Z*-scores by subtracting the global mean and dividing the global standard deviation. Finally, the *z*-transformed ALFF images were smoothed using a 4-mm Gaussian kernel.

*Degree centrality (DC)*

Voxel-wise DC maps were generated using the DPARSF toolbox. For each participant, Pearson’s correlation coefficients were calculated between a given voxel and all other voxels in the brain, resulting in a whole-brain FC matrix. We restricted our analysis to positive correlations above a threshold of *r* = 0.25 to eliminate such voxels having low correlation due to signal noise [4, 5]. That is, for a given voxel, the number of voxels where counted where the correlation between that voxel and another voxel’s BOLD time series exceeded the fixed threshold (i.e., *r* > 0.25). Subsequently, these maps were *z*-transformed and smoothed using a 4-mm FWHM Gaussian kernel.

**References:**

1. Power JD et al. Methods to detect, characterize, and remove motion artifact in resting state fMRI. *Neuroimage* 2014; **84:** 320-341.

2. Murphy K, Birn RM, Handwerker DA, Jones TB, Bandettini PA. The impact of global signal regression on resting state correlations: are anti-correlated networks introduced? *Neuroimage* 2009; **44**(3)**:** 893-905.

3. Yan CG, Craddock RC, Zuo XN, Zang YF, Milham MP. Standardizing the intrinsic brain: towards robust measurement of inter-individual variation in 1000 functional connectomes. *Neuroimage* 2013; **80:** 246-262.

4. Gao C et al. Decreased Subcortical and Increased Cortical Degree Centrality in a Nonclinical College Student Sample with Subclinical Depressive Symptoms: A Resting-State fMRI Study. *Front Hum Neurosci* 2016; **10:** 617.

5. Buckner RL et al. Cortical hubs revealed by intrinsic functional connectivity: mapping, assessment of stability, and relation to Alzheimer's disease. *J Neurosci* 2009; **29**(6)**:** 1860-1873.
