# Supplementary material for: Functional network alterations differently associated with suicidal ideas and acts in depressed patients: an indirect support to the transition model
Source: Transl Psychiatry. 2021 Feb 4;11:100. doi: 10.1038/s41398-021-01232-x (PMC7862288; doi:10.1038/s41398-021-01232-x)

**Supplementary figure S1:**

1. Distribution of depressed patients on the HAM-D item #3 regarding suicidal ideation.
2. Distribution of depressed patients on the BDI item #9 regarding suicidal ideation.

Abbr.: HAM-D, Hamilton depression scale; BDI, Beck Depression Inventory

**A**


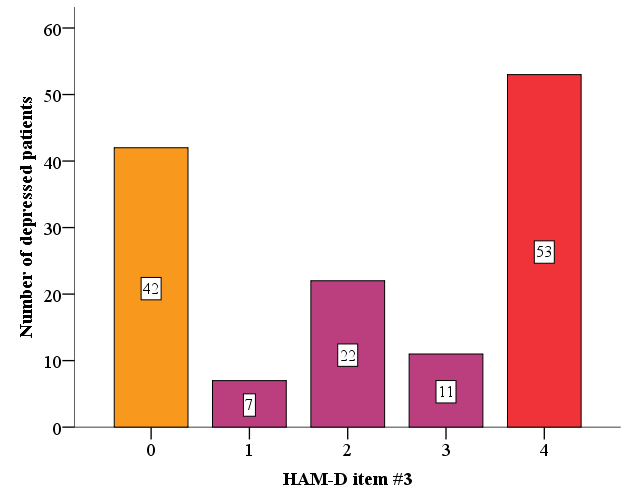


**B**


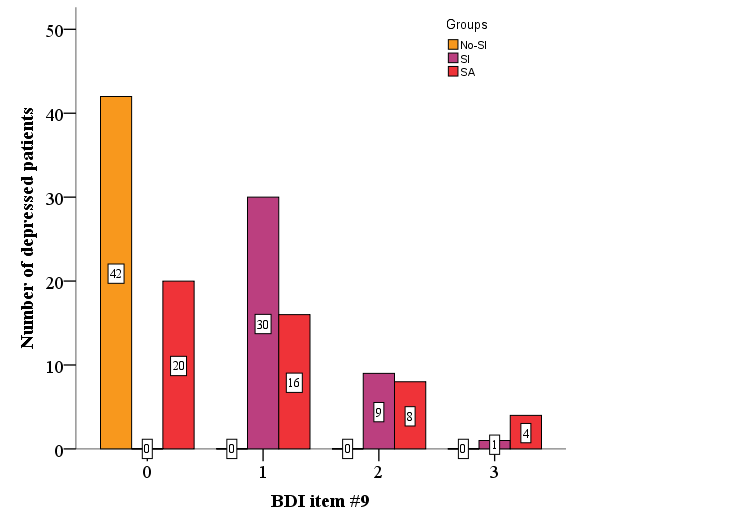

Supplement: Supplementary file 2 — Supplementary Figure S1 [file 41398_2021_1232_MOESM2_ESM.docx]
