# Supplementary material for: Functional network alterations differently associated with suicidal ideas and acts in depressed patients: an indirect support to the transition model
Source: Transl Psychiatry. 2021 Feb 4;11:100. doi: 10.1038/s41398-021-01232-x (PMC7862288; doi:10.1038/s41398-021-01232-x)

**Supplementary figure S2:** Main effect of site (voxel-level FWE-corrected p < 0.05 and cluster-level FDR-corrected p < 0.05) in Amplitude of Low Frequency fluctuations (ALFF) based on healthy subjects from the three different sites, which constituted the largest sample (Stanford: n=36; Jena: n=29 and Montreal: n= 42).


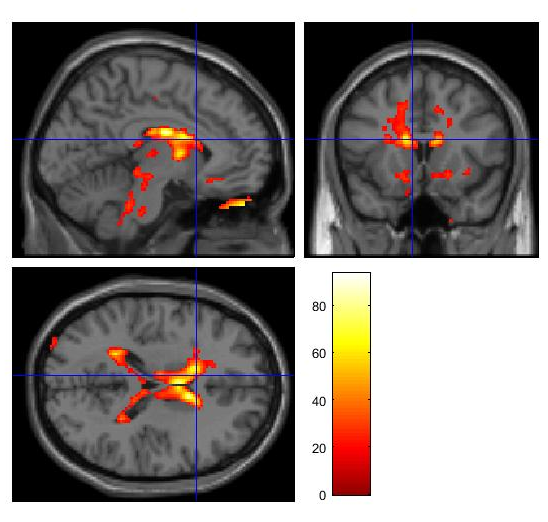

Supplement: Supplementary file 3 — Supplementary Figure S2 [file 41398_2021_1232_MOESM3_ESM.docx]
