# Supplementary material for: Functional network alterations differently associated with suicidal ideas and acts in depressed patients: an indirect support to the transition model
Source: Transl Psychiatry. 2021 Feb 4;11:100. doi: 10.1038/s41398-021-01232-x (PMC7862288; doi:10.1038/s41398-021-01232-x)
Supplement: Supplementary file 4 — Supplementary Table S1 [file 41398_2021_1232_MOESM4_ESM.docx]

**Table S1:** Detailed results of the post-hoc comparisons between SA and both patient groups as well as healthy controls (voxel-level uncorrected p < 0.001, cluster size > 10) on ALFF values, which were significant in the main effect of *group* contrast.

| **ALFF: Healthy controls vs. Patients with SA (p< 0.001, uncorr.)** | | | | | | | | | | | | | | | | | | | |
| --- | --- | --- | --- | --- | --- | --- | --- | --- | --- | --- | --- | --- | --- | --- | --- | --- | --- | --- | --- |
| **Region of activation** | | **Right/Left** | | **Brodmann's Area** | | **Cluster size** | | **MNI coordinates** | | | | | | **T value** | | **p_FWE corr._** | | **q_FDR corr._** | |
|  | |  | |  | |  | | **x** | | **y** | | **z** | |  | |  | |  | |
| Supramarginal gyrus | | L | | 40 | | 31 | | -56 | | -42 | | 38 | | 7.4 | | < 0.001 | | < 0.001 | |
| Angular Gyrus | | R | | 39 | | 74 | | 50 | | -54 | | 34 | | 7.4 | | < 0.001 | | < 0.001 | |
| Angular Gyrus | | R | | 39 | | 57 | | 50 | | -44 | | 44 | | 7.3 | | < 0.001 | | < 0.001 | |
| Angular Gyrus | | L | | 39 | | 40 | | -46 | | -62 | | 32 | | 7.1 | | < 0.001 | | < 0.001 | |
| Paracentral lobule | | R | | 4 | | 13 | | 6 | | -28 | | 66 | | 6.9 | | < 0.001 | | < 0.001 | |
| Inferior frontal gyrus | | R | | 44 | | 14 | | 54 | | 14 | | 10 | | 6.9 | | < 0.001 | | < 0.001 | |
| Supramarginal gyrus | | L | | 40 | | 11 | | -58 | | -50 | | 24 | | 6.9 | | < 0.001 | | < 0.001 | |
| Temporal cortex | | R | | 22 | | 42 | | 58 | | -30 | | 16 | | 6.8 | | < 0.001 | | < 0.001 | |
| Cuneus | | R | | 17 | | 33 | | 0 | | -86 | | 18 | | 6.8 | | < 0.001 | | < 0.001 | |
| Temporal cortex | | L | | 22 | | 15 | | -58 | | -40 | | 20 | | 6.4 | | < 0.001 | | < 0.001 | |
| Angular Gyrus | | L | | 39 | | 47 | | -50 | | -52 | | 36 | | 6.2 | | < 0.001 | | < 0.001 | |
| Inferior parietal cortex | | L | | 40 | | 49 | | -48 | | -36 | | 40 | | 6.2 | | < 0.001 | | < 0.001 | |
| Superior parietal cortex | | R | | 7 | | 54 | | 18 | | -44 | | 68 | | 6.1 | | < 0.001 | | < 0.001 | |
| Supramarginal gyrus | | R | | 40 | | 19 | | 60 | | -32 | | 44 | | 6.1 | | 0.001 | | < 0.001 | |
| Occipital cortex | | L | | 19 | | 16 | | 46 | | -72 | | 12 | | 5.6 | | 0.005 | | 0.001 | |
| Middle frontal gyrus | | R | | 9 | | 32 | | 50 | | 24 | | 22 | | 5.5 | | 0.009 | | 0.002 | |
| Cuneus | | R | | 18 | | 19 | | 6 | | -82 | | 32 | | 5.3 | | 0.022 | | 0.004 | |
| Occipital cortex | | L | | 19 | | 12 | | -38 | | -70 | | 24 | | 5.2 | | 0.026 | | 0.004 | |
| Superior parietal cortex | | R | | 7 | | 13 | | 24 | | -58 | | 64 | | 5.1 | | 0.046 | | 0.007 | |
| Precuneus | | R | | 7 | | 29 | | 14 | | -70 | | 48 | | 5 | | 0.08 | | 0.01 | |
| Superior parietal cortex | | R | | 7 | | 10 | | 26 | | -74 | | 38 | | 4.9 | | 0.092 | | 0.011 | |
| Angular Gyrus | | L | | 39 | | 19 | | -28 | | -60 | | 40 | | 4.5 | | 0.425 | | 0.038 | |
|  | |  | |  | |  | |  | |  | |  | |  | |  | |  | |
| **ALFF: Patients controls vs. Patients with SA (p< 0.001, uncorr.)** | | | | | | | | | | | | | | | | | | | |
| **Region of activation** | | **Right/Left** | | **Brodmann's Area** | | **Cluster size** | | **MNI coordinates** | | | | | | **T value** | | **p_FWE corr._** | | **q_FDR corr._** | |
|  | |  | |  | |  | | **x** | | **y** | | **z** | |  | |  | |  | |
| Superior parietal cortex | | R | | 7 | | 46 | | 18 | | -66 | | 44 | | 8.1 | | < 0.001 | | < 0.001 | |
| Angular Gyrus | | R | | 39 | | 74 | | 50 | | -56 | | 36 | | 7.6 | | < 0.001 | | < 0.001 | |
| Cuneus | | R | | 18 | | 20 | | 4 | | -80 | | 32 | | 7.2 | | < 0.001 | | < 0.001 | |
| Angular Gyrus | | L | | 39 | | 22 | | -26 | | -64 | | 46 | | 7.1 | | < 0.001 | | < 0.001 | |
| Superior parietal cortex | | R | | 7 | | 21 | | 20 | | -60 | | 64 | | 6.5 | | < 0.001 | | < 0.001 | |
| Inferior frontal gyrus | | R | | 9/45 | | 32 | | 50 | | 24 | | 16 | | 6.5 | | < 0.001 | | < 0.001 | |
| Superior parietal cortex | | R | | 7 | | 54 | | 20 | | -44 | | 66 | | 6.5 | | < 0.001 | | < 0.001 | |
| Angular Gyrus | | L | | 39 | | 36 | | -44 | | -60 | | 40 | | 6.5 | | < 0.001 | | < 0.001 | |
| Superior parietal cortex | | L | | 7 | | 11 | | -14 | | -66 | | 58 | | 6.3 | | < 0.001 | | < 0.001 | |
| Occipital cortex | | R | | 19 | | 14 | | 22 | | -80 | | 38 | | 6.4 | | < 0.001 | | < 0.001 | |
| Supramarginal gyrus | | L | | 40 | | 31 | | -56 | | -44 | | 32 | | 6.1 | | < 0.001 | | < 0.001 | |
| Angular Gyrus | | R | | 39 | | 57 | | 54 | | -46 | | 40 | | 6.0 | | 0.017 | | 0.001 | |
| Supramarginal gyrus | | L | | 40 | | 47 | | -52 | | -54 | | 36 | | 6.0 | | 0.028 | | 0.001 | |
| Occipital cortex | | L | | 19 | | 16 | | -40 | | -74 | | 24 | | 6.0 | | 0.001 | | 0.001 | |
| Cuneus | | R | | 18 | | 33 | | 8 | | -86 | | 16 | | 5.8 | | 0.002 | | 0.001 | |
| Occipital cortex | | R | | 19 | | 16 | | 44 | | -70 | | 12 | | 5.7 | | 0.003 | | 0.002 | |
| Supramarginal gyrus | | R | | 40 | | 19 | | 60 | | -30 | | 40 | | 5.7 | | 0.004 | | 0.002 | |
| Supramarginal gyrus | | L | | 40 | | 49 | | -58 | | -32 | | 44 | | 5.49 | | 0.009 | | 0.003 | |
| Supramarginal gyrus | | R | | 40 | | 38 | | 62 | | -34 | | 30 | | 5.33 | | 0.018 | | 0.004 | |
| Supramarginal gyrus | | L | | 40 | | 15 | | -56 | | -38 | | 22 | | 5.1 | | 0.047 | | 0.008 | |
| Angular Gyrus | | L | | 39 | | 11 | | -56 | | -54 | | 24 | | 4.69 | | 0.221 | | 0.026 | |
| Precentral gyrus | | R | | 4 | | 11 | | 6 | | -24 | | 68 | | 4.2 | | 0.258 | | 0.716 | |
| Inferior frontal gyrus | | R | | 44 | | 12 | | 54 | | 16 | | 10 | | 3.56 | | 1.000 | | 0.42 | |
| **ALFF: Patients with SI vs. Patients with SA (p< 0.001, uncorr.)** | | | | | | | | | | | | | | | | | | |  |
| **Region of activation** | **Right/Left** | | **Brodmann's Area** | | **Cluster size** | | **MNI coordinates** | | | | | | **T value** | | **p_FWE corr._** | | **q_FDR corr._** | |  |
|  |  | |  | |  | | **x** | | **y** | | **z** | |  | |  | |  | |  |
| Angular Gyrus | L | | 39 | | 38 | | -46 | | -62 | | 32 | | 6.62 | | 0.045 | | < 0.001 | |  |
| Inferior parietal cortex | L | | 40 | | 28 | | -48 | | -32 | | 36 | | 5.9 | | 0.001 | | 0.005 | |  |
| Occipital cortex | L | | 19 | | 16 | | -38 | | -74 | | 24 | | 5.8 | | 0.002 | | 0.005 | |  |
| Angular Gyrus | R | | 39 | | 63 | | 50 | | -54 | | 34 | | 5.7 | | 0.004 | | 0.006 | |  |
| Precuneus | R | | 7 | | 40 | | 14 | | -70 | | 46 | | 5.5 | | 0.007 | | 0.007 | |  |
| Supramarginal gyrus | R | | 40 | | 49 | | 56 | | -44 | | 38 | | 5.5 | | 0.026 | | 0.008 | |  |
| Superior parietal cortex | R | | 7 | | 21 | | 24 | | -58 | | 64 | | 5.5 | | 0.010 | | 0.008 | |  |
| Postcentral gyrus | R | | 5 | | 53 | | 18 | | -44 | | 72 | | 5.3 | | 0.020 | | 0.010 | |  |
| Paracentral lobule | R | | 4 | | 13 | | 6 | | -26 | | 68 | | 5.1 | | 0.051 | | 0.018 | |  |
| Cuneus | L | | 17 | | 20 | | -4 | | -86 | | 16 | | 4.9 | | 0.116 | | 0.028 | |  |
| Angular Gyrus | L | | 39 | | 45 | | -48 | | -50 | | 36 | | 4.76 | | 0.174 | | 0.036 | |  |
| Occipital cortex | R | | 19 | | 10 | | 26 | | -72 | | 36 | | 4.7 | | 0.190 | | 0.038 | |  |
| Middle frontal gyrus | R | | 9 | | 27 | | 50 | | 24 | | 22 | | 4.4 | | 0.490 | | 0.079 | |  |
| Superior parietal cortex | L | | 7 | | 19 | | -24 | | -62 | | 46 | | 4.05 | | 0.909 | | 0.168 | |  |
| Supramarginal gyrus | R | | 40 | | 13 | | 62 | | -36 | | 28 | | 3.9 | | 0.971 | | 0.215 | |  |
| Cuneus | R | | 18 | | 10 | | 4 | | -80 | | 32 | | 3.8 | | 1.000 | | 0.464 | |  |
| Angular Gyrus | L | | 39 | | 13 | | -58 | | -42 | | 36 | | 3.5 | | 1.000 | | 0.484 | |  |
|  |  | |  | |  | |  | |  | |  | |  | |  | |  | |  |
|  |  | |  | |  | |  | |  | |  | |  | |  | |  | |  |
| **ALFF: patients with SA vs. Healthy controls (p< 0.001, uncorr.)** | | | | | | | | | | | | | | | | | | |  |
| **Region of activation** | **Right/Left** | | **Brodmann's Area** | | **Cluster size** | | **MNI coordinates** | | | | | | **T value** | | **p_FWE corr._** | | **q_FDR corr._** | |  |
|  |  | |  | |  | | **x** | | **y** | | **z** | |  | |  | |  | |  |
| Hippocampus | R | |  | | 102 | | 34 | | -20 | | -20 | | 7.3 | | < 0.001 | | < 0.001 | |  |
| Hippocampus | L | |  | | 48 | | -36 | | -28 | | -26 | | 6.9 | | < 0.001 | | < 0.001 | |  |
| Fusiform gyrus | R | | 20 | | 14 | | 42 | | -14 | | -36 | | 6.3 | | 0.001 | | < 0.001 | |  |
| Thalamus | R | |  | | 10 | | 18 | | -24 | | 20 | | 6.2 | | 0.001 | | < 0.001 | |  |
| Hippocampus | R | |  | | 30 | | 26 | | -22 | | -12 | | 6.2 | | < 0.001 | | < 0.001 | |  |
| Hippocampus | L | |  | | 15 | | -36 | | -14 | | -30 | | 6.0 | | 0.001 | | < 0.001 | |  |
| Hippocampus | L | |  | | 28 | | -24 | | -18 | | -10 | | 5.9 | | 0.001 | | < 0.001 | |  |
|  |  | |  | |  | |  | |  | |  | |  | |  | |  | |  |
| **ALFF: Patients with SA vs. Patient controls (p< 0.001, uncorr.)** | | | | | | | | | | | | | | | | | | |  |
| **Region of activation** | **Right/Left** | | **Brodmann's Area** | | **Cluster size** | | **MNI coordinates** | | | | | | **T value** | | **p_FWE corr._** | | **q_FDR corr._** | |  |
|  |  | |  | |  | | **x** | | **y** | | **z** | |  | |  | |  | |  |
| Fusiform gyrus | L | | 20 | | 15 | | -36 | | -16 | | -30 | | 5.7 | | 0.004 | | 0.013 | |  |
| Hippocampus | R | |  | | 96 | | 32 | | -14 | | -26 | | 5.3 | | 0.020 | | 0.030 | |  |
| Fusiform gyrus | R | | 20 | | 14 | | 42 | | -12 | | -36 | | 5.2 | | 0.031 | | 0.030 | |  |
| Hippocampus | L | |  | | 28 | | -22 | | -22 | | -10 | | 5.09 | | 0.049 | | 0.03 | |  |
| Hippocampus | R | |  | | 30 | | 28 | | -20 | | -12 | | 5.0 | | 0.066 | | 0.030 | |  |
| Hippocampus | L | |  | | 47 | | -38 | | -38 | | -38 | | 4.9 | | 0.095 | | 0.033 | |  |
| Thalamus | R | |  | | 10 | | 18 | | -24 | | 22 | | 3.96 | | 0.962 | | 0.214 | |  |
|  |  | |  | |  | |  | |  | |  | |  | |  | |  | |  |
| **ALFF: Patients with SA vs. Patients with SI (p< 0.001, uncorr.)** | | | | | | | | | | | | | | | | | | |  |
| **Region of activation** | **Right/Left** | | **Brodmann's Area** | | **Cluster size** | | **MNI coordinates** | | | | | | **T value** | | **p_FWE corr._** | | **q_FDR corr._** | |  |
|  |  | |  | |  | | **x** | | **y** | | **z** | |  | |  | |  | |  |
| Parahippocampal gyrus | L | | 36 | | 15 | | -36 | | -16 | | -30 | | 5.7 | | 0.004 | | 0.013 | |  |
| Hippocampus | L | |  | | 96 | | -36 | | -16 | | -30 | | 5.3 | | 0.020 | | 0.030 | |  |
| Fusiform gyrus | R | | 20 | | 14 | | 42 | | -12 | | -36 | | 5.2 | | 0.031 | | 0.030 | |  |
| Hippocampus | L | |  | | 28 | | -22 | | -22 | | -10 | | 5.1 | | 0.049 | | 0.030 | |  |
| Hippocampus | R | |  | | 30 | | 28 | | -20 | | -12 | | 5.0 | | 0.066 | | 0.030 | |  |
| Hippocampus | R | |  | | 47 | | -38 | | -34 | | -16 | | 4.9 | | 0.095 | | 0.033 | |  |
| Thalamus | R | |  | | 12 | | 18 | | -24 | | 22 | | 3.96 | | 0.962 | | 0.214 | |  |
|  |  | |  | |  | |  | |  | |  | |  | |  | |  | |  |
