# Supplementary material for: Functional network alterations differently associated with suicidal ideas and acts in depressed patients: an indirect support to the transition model
Source: Transl Psychiatry. 2021 Feb 4;11:100. doi: 10.1038/s41398-021-01232-x (PMC7862288; doi:10.1038/s41398-021-01232-x)
Supplement: Supplementary file 5 — Supplementary Table S2 [file 41398_2021_1232_MOESM5_ESM.docx]

**Table S2:** Detailed results of the post-hoc comparisons between patient controls and healthy controls as well as patients with SI (voxel-level uncorrected p < 0.001, cluster size >10) on ALFF values, which were significant in the main effect of *group* contrast.

| **ALFF: Patients controls vs. Healthy controls (p< 0.001, uncorr.)** | | | | | | | | | |
| --- | --- | --- | --- | --- | --- | --- | --- | --- | --- |
| **Region of activation** | **Right/Left** | **Brodmann's Area** | **Cluster size** | **MNI coordinates** | | | **T value** | **p_FWE corr._** | **q_FDR corr._** |
|  |  |  |  | **x** | **y** | **z** |  |  |  |
| Superior parietal cortex | R | 7 | 40 | 18 | -66 | 42 | 6.4 | < 0.001 | < 0.001 |
| Superior parietal cortex | L | 7 | 11 | -16 | -64 | 56 | 5.1 | 0.044 | 0.059 |
| Superior parietal cortex | R | 7 | 13 | 20 | -60 | 64 | 5.0 | 0.066 | 0.067 |
| Superior parietal cortex | L | 7 | 20 | -26 | -64 | 46 | 4.7 | 0.208 | 0.153 |
| Occipital cortex | R | 19 | 10 | 22 | -80 | 40 | 4.5 | 0.452 | 0.281 |
| Cuneus | R | 17 | 10 | 4 | -80 | 32 | 4.1 | 0.882 | 0.298 |
| Middle frontal gyrus | R | 9 | 10 | 54 | 26 | 18 | 3.88 | 0.984 | 0.359 |
|  |  |  |  |  |  |  |  |  |  |
| **ALFF: Patients controls vs. Patients with SI (p< 0.001, uncorr.)** | | | | | | | | | |
| **Region of activation** | **Right/Left** | **Brodmann's Area** | **Cluster size** | **MNI coordinates** | | | **T value** | **p_FWE corr._** | **q_FDR corr._** |
|  |  |  |  | **x** | **y** | **z** |  |  |  |
| Superior parietal cortex | R | 7 | 15 | 18 | -64 | 42 | 4.5 | 0.446 | 0.704 |
