# Supplementary material for: Functional network alterations differently associated with suicidal ideas and acts in depressed patients: an indirect support to the transition model
Source: Transl Psychiatry. 2021 Feb 4;11:100. doi: 10.1038/s41398-021-01232-x (PMC7862288; doi:10.1038/s41398-021-01232-x)
Supplement: Supplementary file 6 — Supplementary Table S3 [file 41398_2021_1232_MOESM6_ESM.docx]

**Table S3.** Detailed results of the post-hoc comparisons between groups (voxel-level uncorrected p < 0.001, cluster extent > 10 voxels) on DC values, which were significant in the main effect of *group* contrast.

| **DC: Healthy controls vs. Patients with SA (p< 0.001, uncorr.)** | | | | | | | | | |
| --- | --- | --- | --- | --- | --- | --- | --- | --- | --- |
| **Region of activation** | **Right/Left** | **Brodmann's Area** | **Cluster size** | **MNI coordinates** | | | **T value** | **p_FWE corr._** | **q_FDR corr._** |
|  |  |  |  | **x** | **y** | **z** |  |  |  |
| Angular Gyrus | L | 39 | 36 | -42 | -60 | 10 | 7.1 | < 0.001 | < 0.001 |
| Occipital cortex | L | 18 | 14 | -14 | -72 | 0 | 6.8 | < 0.001 | < 0.001 |
| Occipital cortex | L | 19 | 12 | -10 | -84 | 26 | 6.1 | < 0.001 | 0.001 |
| Occipital cortex | R | 18 | 23 | 14 | -80 | 20 | 5.9 | 0.001 | 0.001 |
| Angular Gyrus | R | 39 | 16 | 46 | -54 | 38 | 5.8 | 0.002 | 0.001 |
| Inferior frontal gyrus | R | 9/45 | 11 | 50 | 24 | 14 | 5.1 | 0.04 | 0.008 |
|  |  |  |  |  |  |  |  |  |  |
| **DC: Patient controls vs. Patients with SA (p< 0.001, uncorr.)** | | | | | | | | | |
| **Region of activation** | **Right/Left** | **Brodmann's Area** | **Cluster size** | **MNI coordinates** | | | **T value** | **p_FWE corr._** | **q_FDR corr._** |
|  |  |  |  | **x** | **y** | **z** |  |  |  |
| Superior parietal cortex | R | 7 | 31 | 18 | -64 | 42 | 7.4 | < 0.001 | < 0.001 |
| Inferior frontal gyrus | R | 9/45 | 11 | 52 | 24 | 16 | 6.1 | < 0.001 | 0.001 |
| Occipital cortex | R | 18 | 23 | 14 | -82 | 18 | 6.1 | < 0.001 | 0.001 |
| Angular Gyrus | L | 39 | 28 | -44 | -58 | 6 | 6.07 | 0.001 | 0.001 |
| Angular Gyrus | R | 39 | 16 | 44 | -56 | 40 | 6.0 | 0.001 | 0.001 |
| Occipital cortex | L | 18 | 11 | -14 | -72 | 0 | 4.91 | 0.102 | 0.021 |
|  |  |  |  |  |  |  |  |  |  |
| **DC: Patients with SI vs. Patients with SA (p< 0.001, uncorr.)** | | | | | | | | | |
| **Region of activation** | **Right/Left** | **Brodmann's Area** | **Cluster size** | **MNI coordinates** | | | **T value** | **p_FWE corr._** | **q_FDR corr._** |
|  |  |  |  | **x** | **y** | **z** |  |  |  |
| Occipital cortex | L | 18 | 14 | -14 | -74 | -2 | 6.0 | 0.001 | 0.004 |
| Inferior frontal gyrus | R | 9/45 | 10 | 48 | 24 | 12 | 5.8 | 0.003 | 0.005 |
| Angular Gyrus | R | 39 | 16 | 46 | -54 | 38 | 5.8 | 0.002 | 0.005 |
| Superior parietal cortex | R | 7 | 21 | 22 | -68 | 42 | 5.1 | 0.052 | 0.023 |
|  |  |  |  |  |  |  |  |  |  |
| **DC: Patient controls vs. Healthy controls (p< 0.001, uncorr.)** | | | | | | | | | |
| **Region of activation** | **Right/Left** | **Brodmann's Area** | **Cluster size** | **MNI coordinates** | | | **T value** | **p_FWE corr._** | **q_FDR corr._** |
|  |  |  |  | **x** | **y** | **z** |  |  |  |
| Superior parietal cortex | R | 7 | 31 | 18 | -66 | 42 | 8.1 | < 0.001 | < 0.001 |
|  |  |  |  |  |  |  |  |  |  |
|  |  |  |  |  |  |  |  |  |  |
| **DC: Patient controls vs. Patients with SI (p< 0.001, uncorr.)** | | | | | | | | | |
| **Region of activation** | **Right/Left** | **Brodmann's Area** | **Cluster size** | **MNI coordinates** | | | **T value** | **p_FWE corr._** | **q_FDR corr._** |
|  |  |  |  | **x** | **y** | **z** |  |  |  |
| Occipital cortex | R | 18 | 10 | 20 | -78 | 20 | 5.1 | 0.420 | 0.220 |
|  |  |  |  |  |  |  |  |  |  |
| **DC: Healthy controls vs. Patients with SI (p< 0.001, uncorr.)** | | | | | | | | | |
| **Region of activation** | **Right/Left** | **Brodmann's Area** | **Cluster size** | **MNI coordinates** | | | **T value** | **p_FWE corr._** | **q_FDR corr._** |
|  |  |  |  | **x** | **y** | **z** |  |  |  |
| Angular Gyrus | L | 39 | 18 | -42 | -62 | 10 | 5.1 | 0.040 | 0.050 |
|  |  |  |  |  |  |  |  |  |  |
| **DC: Patients with SI vs. Healthy controls (p< 0.001, uncorr.)** | | | | | | | | | |
| **Region of activation** | **Right/Left** | **Brodmann's Area** | **Cluster size** | **MNI coordinates** | | | **T value** | **p_FWE corr._** | **q_FDR corr._** |
|  |  |  |  | **x** | **y** | **z** |  |  |  |
| Superior parietal cortex | R | 7 | 17 | 16 | -66 | 44 | 4.3 | 0.623 | 0.272 |
